# Supplementary material for: Omics-Inferred Partitioning and Expression of Diverse Biogeochemical Functions in a Low-O2 Cyanobacterial Mat Community
Source: mSystems. 2021 Dec 7;6(6):e01042-21. doi: 10.1128/mSystems.01042-21 (PMC8651085; doi:10.1128/mSystems.01042-21)
Supplement: TABLE S1 [file msystems.01042-21-st001.pdf]

Table S1. Summary of samples and accession numbers for NCBI Short Read Archive (SRA)

Seq ID	Type	Time	gDNA reads	cDNA reads	Accession
MISgDNA_2007-1D	finger	day	55,742,528	-	SRS455884
MISgDNA_2009-1D	prostrate	day	56,610,020	-	SRS455885
MISgDNA_2009-2D	finger	day	58,811,950	-	SRS455886
MISgDNA_2009-3D	finger	day	25,542,490	-	SRS455906
MISgDNA_2010-1D	finger	day	69,716,858	-	SRS455888
MISgDNA_2010-2D	prostrate	day	67,190,598	-	SRR15016858
MISgDNA_2011-1D	prostrate	day	98,430,946	-	SRS455889
MISgDNA_2011-2N	prostrate	night	211,878,358	-	SRS455890
MISgDNA_2011-3D	finger	day	59,608,800	-	SRS455891
MISgDNA_2012-1D	finger	day	25,666,608	-	SRS455892
MISgDNA_2012-2D	finger	day	41,595,684	-	SRS455893
MISgDNA_2012-3D	finger	day	41,318,538	-	SRS455894
MISgDNA_2012-4N	finger	night	42,876,682	-	SRS455895
MISgDNA_2012-5N	finger	night	38,335,454	-	SRS455896
MISgDNA_2012-6N	finger	night	28,207,614	-	SRS455898
MIScDNA_2012-1D	finger	day	-	11,997,534	SRS455900
MIScDNA_2012-2D	finger	day	-	14,577,110	SRS455901
MIScDNA_2012-3D	finger	day	-	11,524,300	SRS455902
MIScDNA_2012-4N	finger	night	-	12,125,414	SRS455903
MIScDNA_2012-5N	finger	night	-	12,100,438	SRS455904
MIScDNA_2012-6N	finger	night	-	13,079,448	SRS455905

[1] "lift-off" mat structure *sensu* Voorhies et al. 2012

[2] flat mat

[3] numbers reflect de-replicated and trimmed read totals used for mapping.
