# Supplementary material for: Omics-Inferred Partitioning and Expression of Diverse Biogeochemical Functions in a Low-O2 Cyanobacterial Mat Community
Source: mSystems. 2021 Dec 7;6(6):e01042-21. doi: 10.1128/mSystems.01042-21 (PMC8651085; doi:10.1128/mSystems.01042-21)

Figure S1. Contig length and taxonomic classification of single copy genes (SCG) from the *Phormidium* bin.

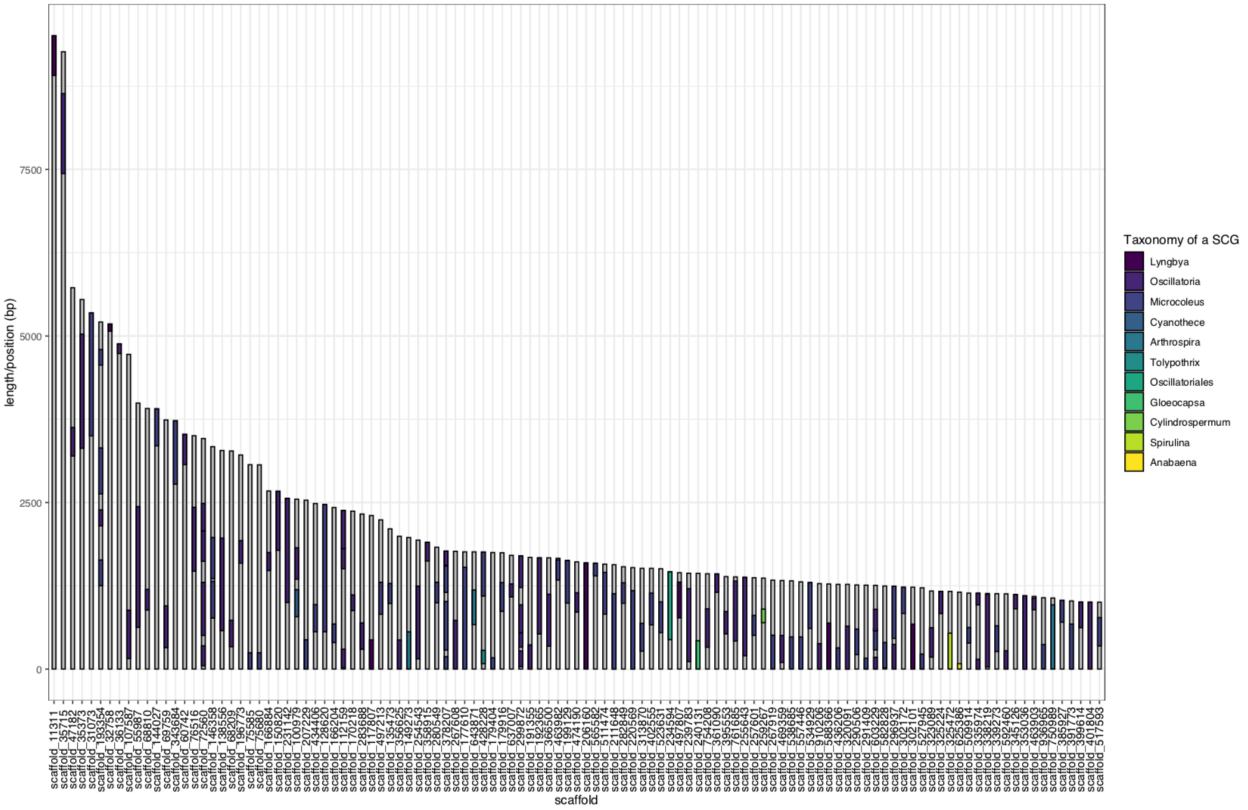

Supplement: FIG S1 [file msystems.01042-21-sf001.pdf]
