# Supplementary material for: Omics-Inferred Partitioning and Expression of Diverse Biogeochemical Functions in a Low-O2 Cyanobacterial Mat Community
Source: mSystems. 2021 Dec 7;6(6):e01042-21. doi: 10.1128/mSystems.01042-21 (PMC8651085; doi:10.1128/mSystems.01042-21)

Figure S2. Average metagenomic (gDNA) and metatranscriptomic (cDNA) coverage of MAGs in each sample. Colors indicate taxonomic designation of MAGs at the genus level, where available. Multiple MAGs may have the same taxonomic designation, and thus share the same color.

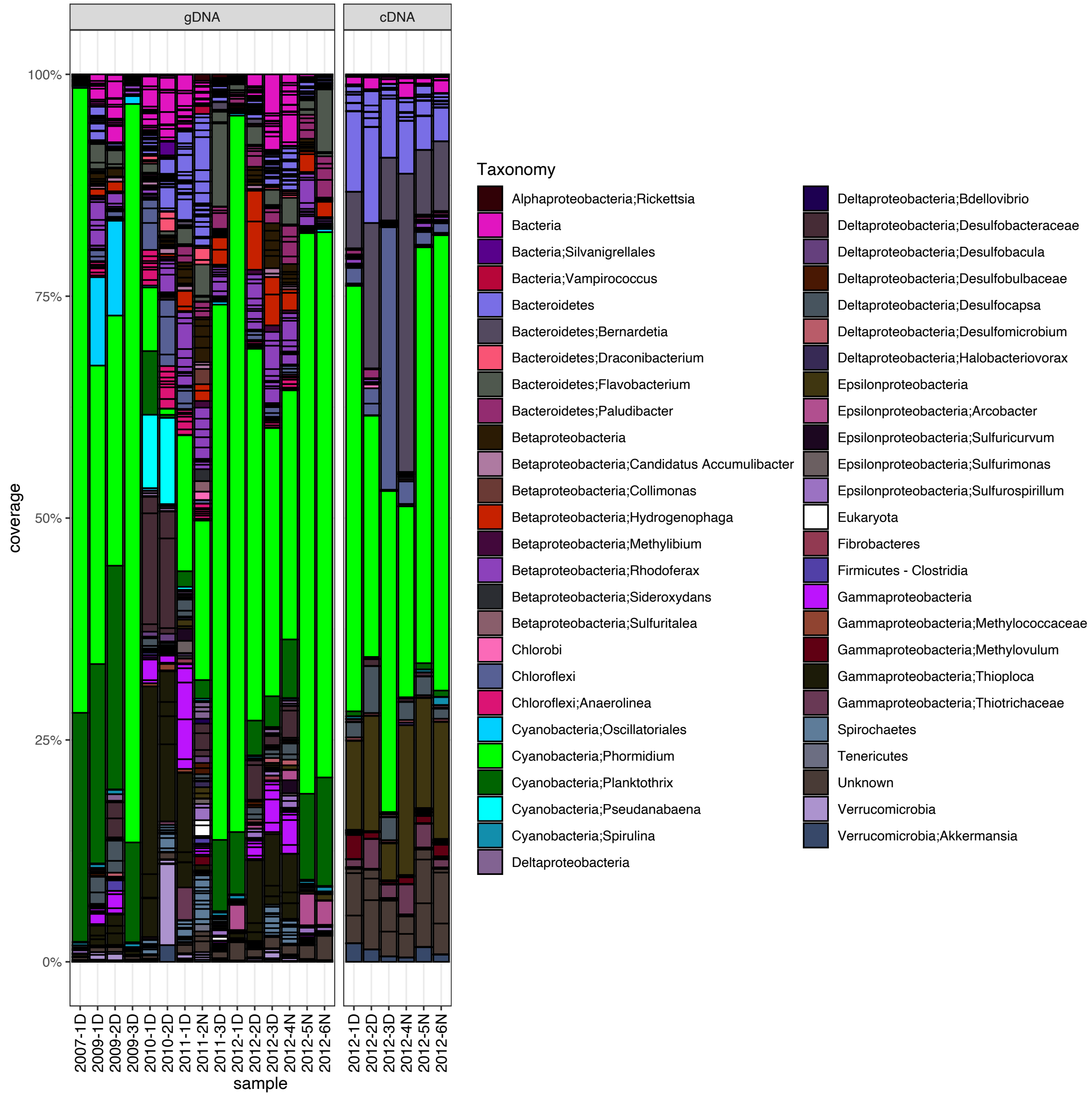

Supplement: FIG S2 [file msystems.01042-21-sf002.pdf]
