# Supplementary material for: Omics-Inferred Partitioning and Expression of Diverse Biogeochemical Functions in a Low-O2 Cyanobacterial Mat Community
Source: mSystems. 2021 Dec 7;6(6):e01042-21. doi: 10.1128/mSystems.01042-21 (PMC8651085; doi:10.1128/mSystems.01042-21)

Figure S3. Average metagenomic (gDNA) and metatranscriptomic (cDNA) coverage of putative diatom chloroplasts. Binned scaffolds in putative diatoms were identified as chloroplasts based on presence of photosynthetic reaction center genes (*psbA*).

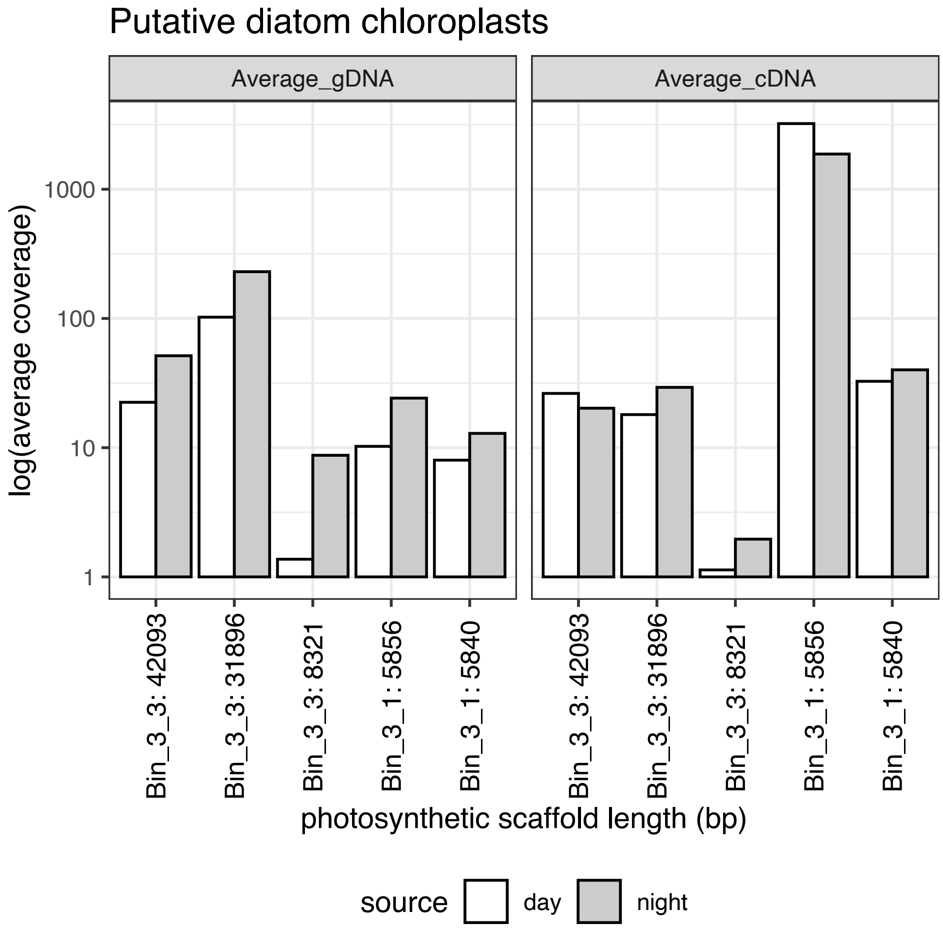

Supplement: FIG S3 [file msystems.01042-21-sf003.pdf]
