# Supplementary material for: Omics-Inferred Partitioning and Expression of Diverse Biogeochemical Functions in a Low-O2 Cyanobacterial Mat Community
Source: mSystems. 2021 Dec 7;6(6):e01042-21. doi: 10.1128/mSystems.01042-21 (PMC8651085; doi:10.1128/mSystems.01042-21)

Figure S4. Relative abundance of transcripts from *psbA* and *psaA* genes in day and night, normalized to the number of transcripts recruited to each MAG. Log-transformed bin-specific TPM of transcript abundance in the day (white) and night (grey) of genes encoding photosystem I (*psaA*) and photosystem II (*psbA*) is shown for each MAG (indicated at top). X-axis labels “psbA_2”, “psbA_3” and “psbA_4” refer to *psbA* types (see text). Boxes represent the 25-75th percentiles, the inside line is the median, and whiskers extend to minimum and maximum values. Observations are overlaid as points.

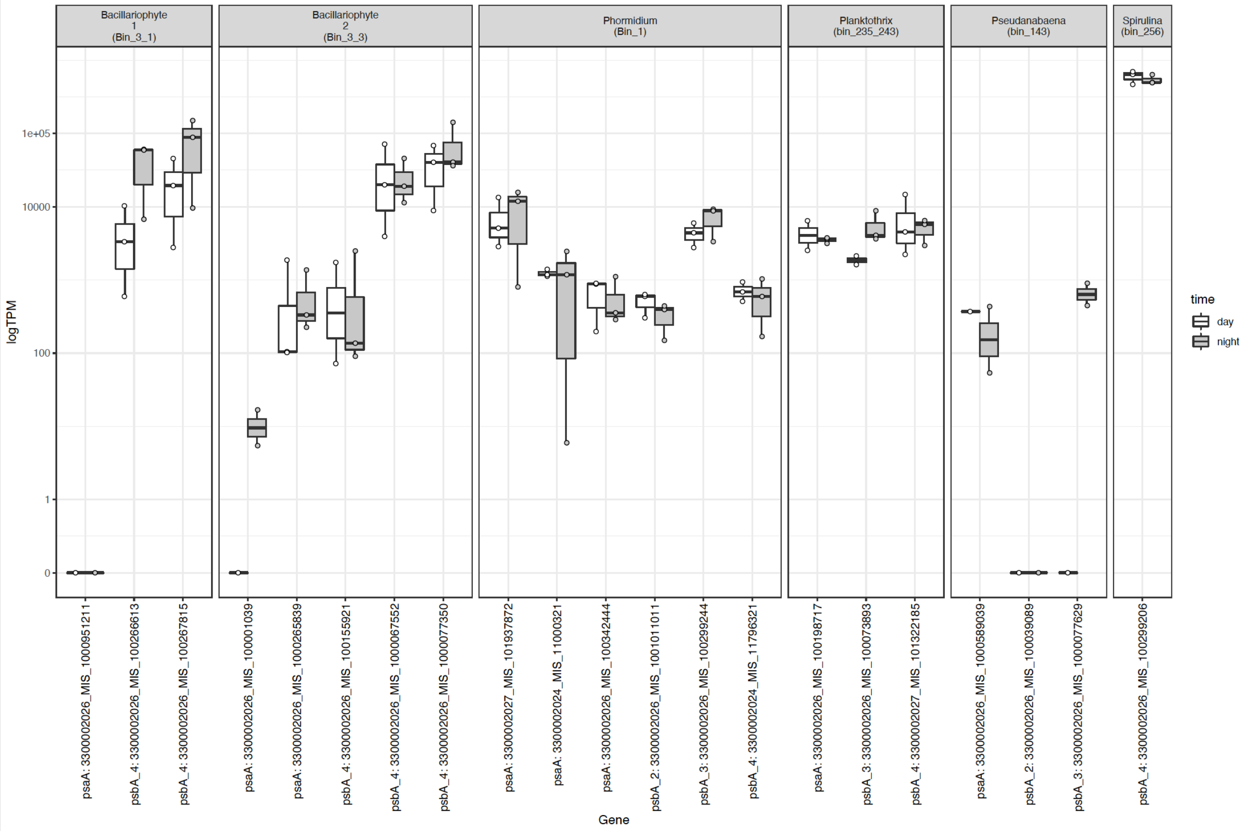

Supplement: FIG S4 [file msystems.01042-21-sf004.pdf]
