# Supplementary material for: Omics-Inferred Partitioning and Expression of Diverse Biogeochemical Functions in a Low-O2 Cyanobacterial Mat Community
Source: mSystems. 2021 Dec 7;6(6):e01042-21. doi: 10.1128/mSystems.01042-21 (PMC8651085; doi:10.1128/mSystems.01042-21)

Figure S5. Relative abundance of transcripts from *sqr*, *psaL*, and *psaX* from the *Phormidium* bin in day and night, normalized to the number of sequences retrieved from each sample. Sample-normalized TPM of transcript abundance in the day (white) and night (grey) is shown as box an whiskers plots in which boxes represent the 25-75th percentiles, the inside line is the median, and whiskers extend to minimum and maximum values. Observations are overlaid as points.

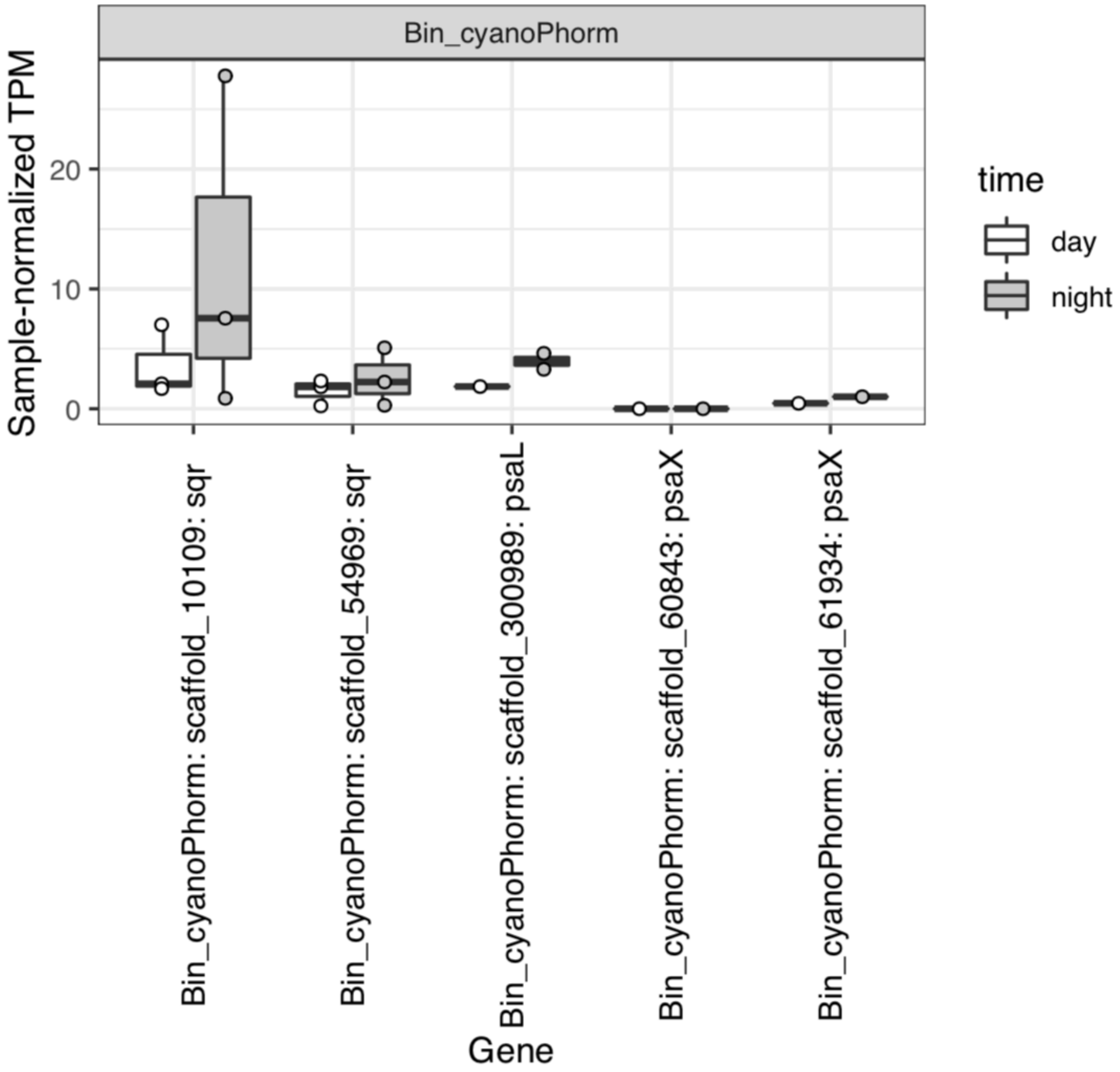

Supplement: FIG S5 [file msystems.01042-21-sf005.pdf]
