# Supplementary material for: Omics-Inferred Partitioning and Expression of Diverse Biogeochemical Functions in a Low-O2 Cyanobacterial Mat Community
Source: mSystems. 2021 Dec 7;6(6):e01042-21. doi: 10.1128/mSystems.01042-21 (PMC8651085; doi:10.1128/mSystems.01042-21)

Figure S6. Relative abundance of transcripts from genes encoding terminal respiratory oxidases in the *Phormidium* MAG, normalized to the number of transcripts recruited to the *Phormidium* MAG in each sample. Log-transformed bin-specific TPM of transcript abundance in the day (white) and night (grey) of genes are shown as box and whiskers plots in which boxes represent the 25-75th percentiles, the inside line is the median, and whiskers extend to minimum and maximum values. Observations are overlaid as points.

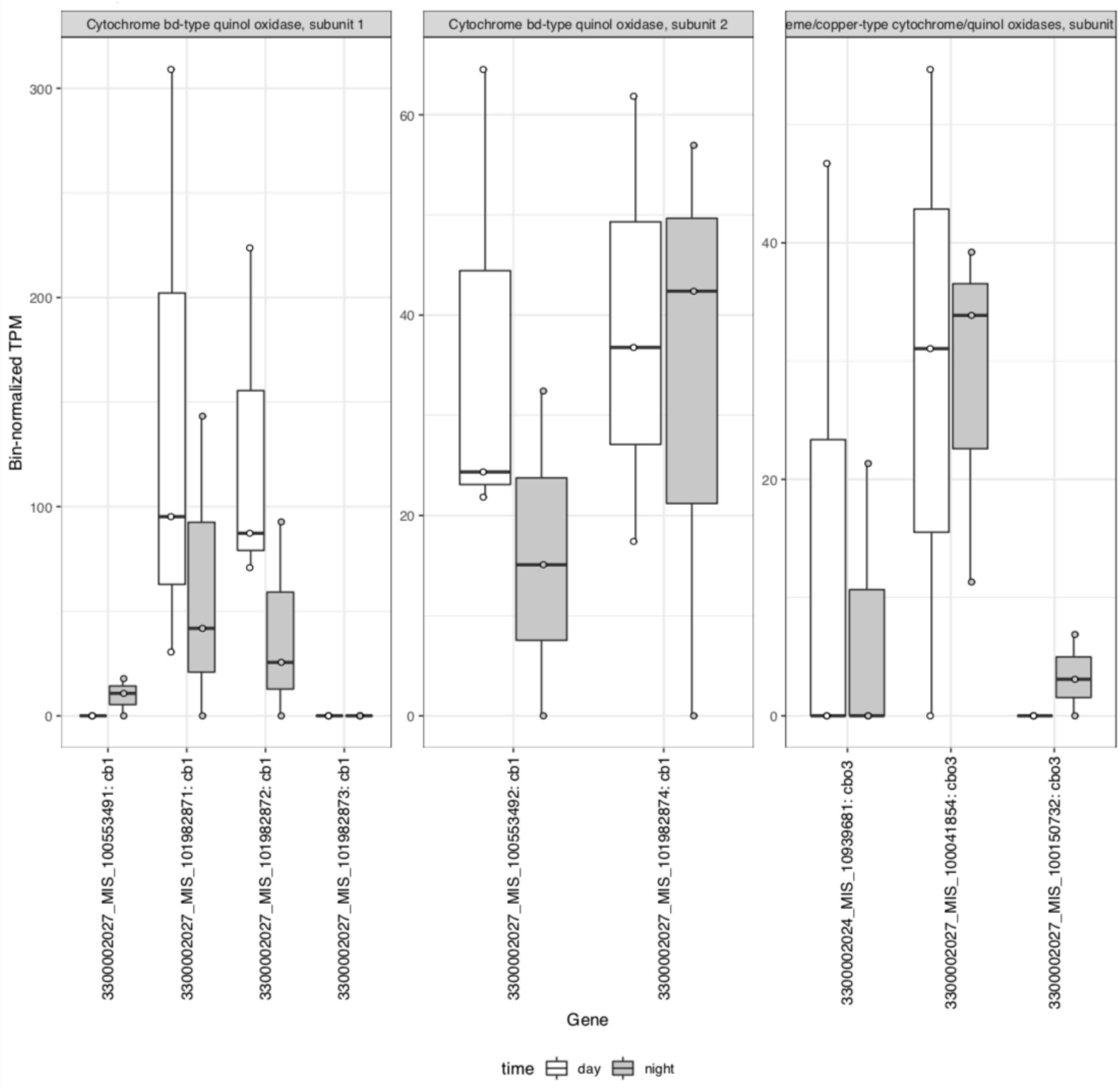

Supplement: FIG S6 [file msystems.01042-21-sf006.pdf]
