# Supplementary material for: Omics-Inferred Partitioning and Expression of Diverse Biogeochemical Functions in a Low-O2 Cyanobacterial Mat Community
Source: mSystems. 2021 Dec 7;6(6):e01042-21. doi: 10.1128/mSystems.01042-21 (PMC8651085; doi:10.1128/mSystems.01042-21)

Figure S7. Transcriptional relative abundance and MAG bin statistics of genes involved in dissimilatory sulfite reduction (dsr, *dsrA* and *dsrD* genes) and reverse dsr (*rdsrA* and *dsrEFH*). In the first and third panels, the values represent sample-normalized TPM, shown as box and whiskers plots in which boxes represent the 25-75th percentiles, the inside line is the median, and whiskers extend to minimum and maximum values. Observations are overlaid as points. The second and fourth panels present the percent completion of MAG bins, when available, in which the genes are located. Many genes were unable to be binned into MAG bins (indicated with NA in label), despite being transcriptionally active in day (white) or night (grey). The average coverage in metatranscriptomic samples as presented in Table S2.

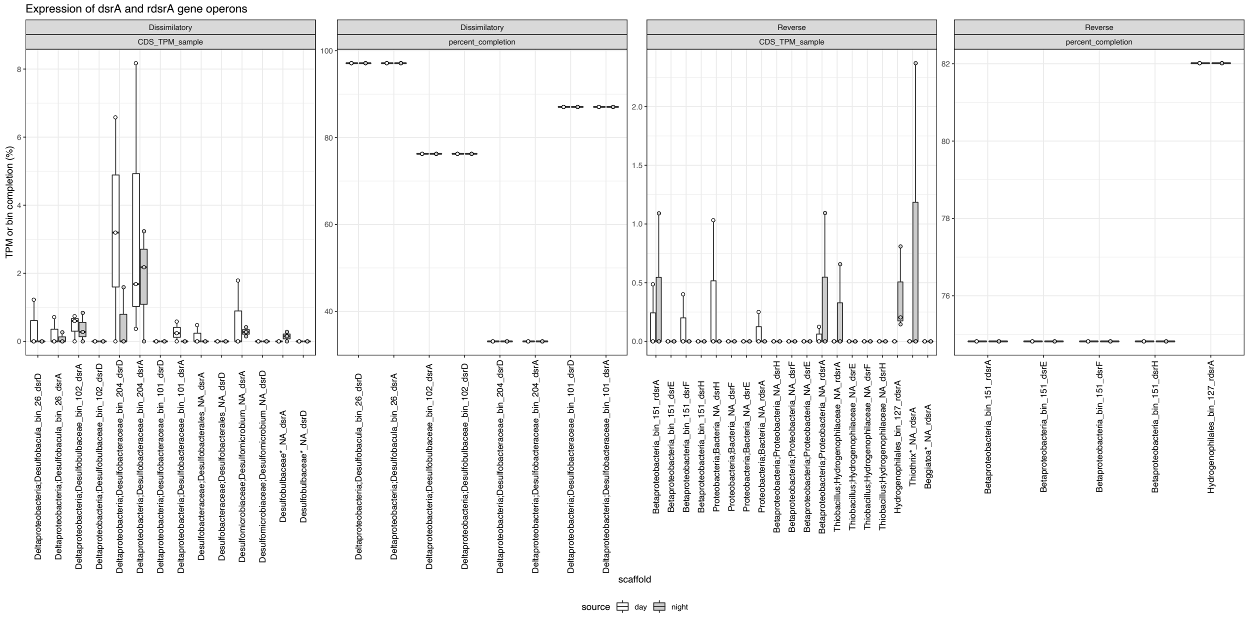

Supplement: FIG S7 [file msystems.01042-21-sf007.pdf]
