# Supplementary material for: Omics-Inferred Partitioning and Expression of Diverse Biogeochemical Functions in a Low-O2 Cyanobacterial Mat Community
Source: mSystems. 2021 Dec 7;6(6):e01042-21. doi: 10.1128/mSystems.01042-21 (PMC8651085; doi:10.1128/mSystems.01042-21)

Figure S8. Abundance of transcripts from genes encoding oxidation of various sulfur species. The sample-normalized TPM of day (white) and night (grey) transcripts of genes *fccAB* for sulfide oxidation using flavocytochrome C sulfide dehydrogenase, and *soxA* for thiosulfate oxidation, are plotted by taxonomy of the MAG (if available) or scaffold (if unbinned). Boxes represent the 25-75th percentiles, the inside line is the median, and whiskers extend to minimum and maximum values. Observations are overlaid as points. Only those MAGs or scaffolds that recruited transcripts are shown here; 40 additional *soxA* genes and 40 additional *fccAB* genes were identified in the metagenomic dataset but were not represented in the metatranscriptome.

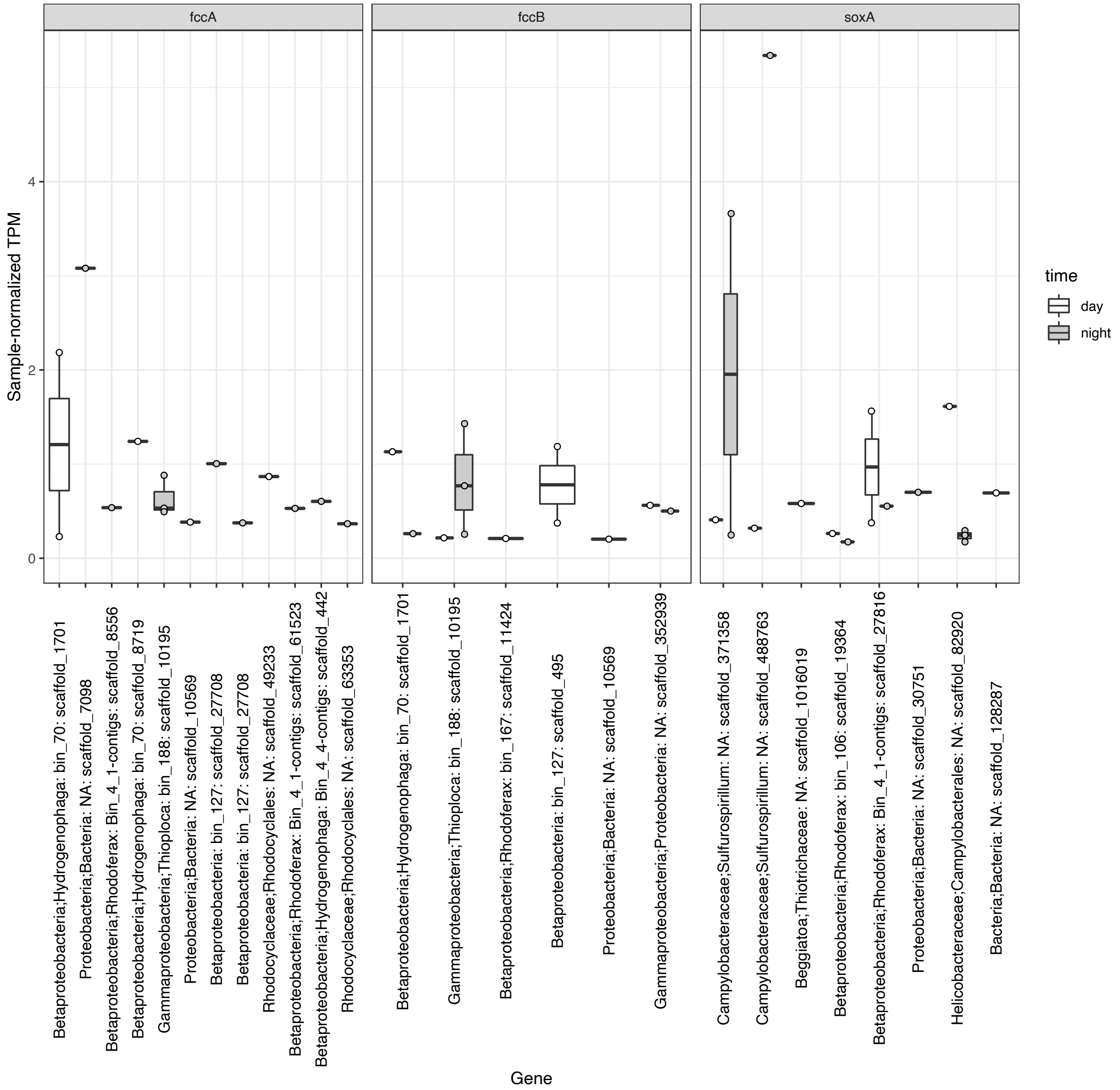

Supplement: FIG S8 [file msystems.01042-21-sf008.pdf]
